# Supplementary material for: The Construction and Exploration of a Comprehensive MicroRNA Centered Regulatory Network in Foxtail Millet (Setaria italica L.)
Source: Front Plant Sci. 2022 May 6;13:848474. doi: 10.3389/fpls.2022.848474 (PMC9121102; doi:10.3389/fpls.2022.848474)
Supplement: Supplementary file 5 [file Data_Sheet_5.zip › Sit-miR398a_Seita.9G403600.1_111_TPlot.pdf]

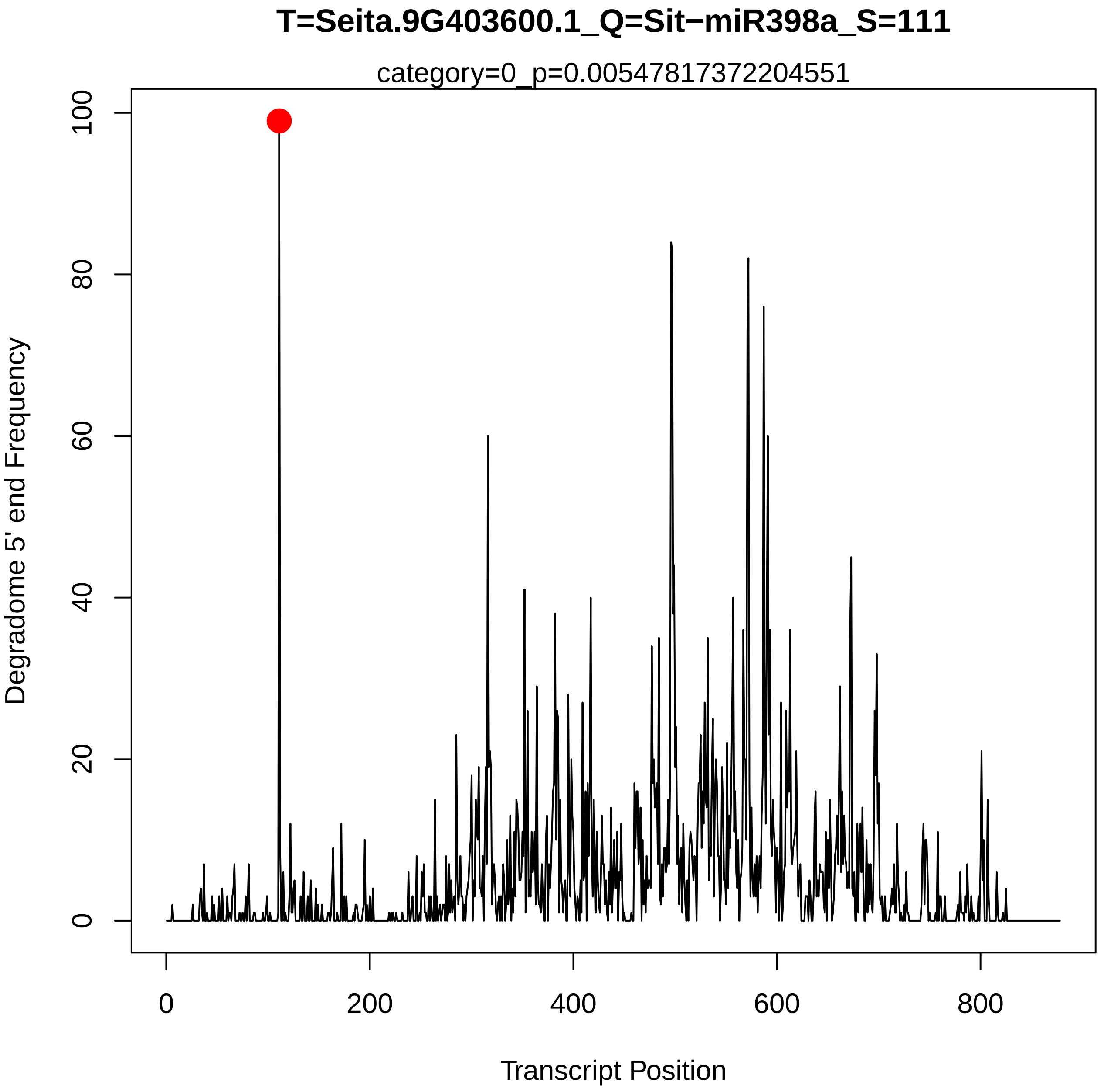
T=Seita.9G403600.1_Q=Sit−miR398a_S=111
category=0_p=0.00547817372204551
Degradome 5' end Frequency
0
20
40
60
80
100
Transcript Position
0
200
400
600
800
